# Epihack Sri Lanka: development of a mobile surveillance tool for dengue fever

**DOI:** 10.1186/s12911-019-0829-5

**Published:** 2019-06-13

**Authors:** May O. Lwin, Anita Sheldenkar, Chitra Panchapakesan, Janelle Shaina Ng, Jerrald Lau, Karthikayen Jayasundar, Kasun Horathalge, Vajira Sampath Rathnayake, Adam W. Crawley, Prasad Wimalaratne

**Affiliations:** 1 Wee Kim Wee School of Communication and Information, Nanyang Technological University (NTU), 31 Nanyang Link, Singapore, 637718 Singapore; 2 SoftVil Technologies (Pvt) Ltd, 423/2C, Old Road, Kottawa, Sri Lanka; 3 Ending Pandemics, 870 Market St, San Francisco, CA 94102 USA; 4 University of Colombo School of Computing, UCSC Building Complex, 35 Reid Ave, Colombo, 00700 Sri Lanka

**Keywords:** Digital surveillance, Dengue, Health communication, Asia, EpiHack

## Abstract

**Background:**

Dengue is a serious problem around the globe, with 3.9 billion people at risk of the disease. Sri Lanka has recently seen unprecedented rates of dengue with 4.3 times more cases than during the same period over the previous six years. The paper discusses the development of an integrated health systems framework, aided by mobile technology, to combat and contain dengue via a health hackathon in Sri Lanka.

**Results:**

The framework addresses the key functions of surveillance, health communication and civic engagement through innovations including digitisation of hospital forms; digital aid to Public Health Inspectors (PHIs); data consolidation and analytics; education for construction workers, GPs, and schools; and educating the general public.

**Conclusions:**

We present the impact of the disease burden in tropical countries, such as Sri Lanka, current technological solutions, and the process of developing the mobile application modules developed via the health hackathon.

## Background

### Dengue background

The incidence of dengue continues to rise across Asia. Approximately 3.9 billion people globally are at risk of dengue [1], and it afflicts approximately 390 million people each year [2]. WHO [3] estimates that given severe underreporting and misclassification of dengue, at least 264 disability adjusted life years per million population per year are lost, which has huge implications for public healthcare systems.

Sri Lanka is a rapidly developing tropical island country located in South Asia affected by with dengue. The nation recently presented nearly 4.3 times more dengue cases than the regular rate of incidence [4]. This led to 215 deaths in the first half of 2017 alone, with the capital city of Colombo having the highest number of reported dengue cases, possibly due to heavy flooding and pooling of stagnant water at numerous construction sites [5]. This provided ample breeding grounds for dengue-carrying mosquitos and magnified the difficulty of government, private, or community efforts to remove these sites.

This paper traces the development of a dengue surveillance application via a unique EpiHack™ event to build the conceptual foundations of an integrated system for the control and eradication of dengue in Sri Lanka. This paper also briefly describes some of the most innovative features of such surveillance and expounds on the process of streamlining inefficient operations in the Colombo public health system. The technical specifics of this application are also illustrated to provide a clearer understanding of its implementation.

### Technological solutions for dengue surveillance

To combat dengue, in recent years public health authorities around the world have adopted new technology and developed and implemented different digital vector surveillance systems where members of the public can participate in some form of active on-the-ground surveillance of mosquitos. Traditional dengue surveillance methods involved using paper based forms and face to face communication. Such digital systems, when developed and utilised well, offer numerous pragmatic benefits to anti-dengue efforts. For example, in Nicaragua, a comprehensive composite mapping system provided the Nicaraguan Ministry of Health with detailed information on potential dengue breeding sites and locations of patients with a history of dengue infection [6]. The researchers found that the map allowed public health workers to identify areas at greater risk of outbreaks. This made it possible to plan vector control strategies and interventions more quickly and more efficiently use public health resources. In terms of key functionalities needed for such technologies, a review by Beatty et al. (2010) identified *detection*, *reporting*, *investigation*, *confirmation*, *analysis*, *interpretation*, and *response*, as commonalities between dengue surveillance systems. They further reported that simplification of case reporting, quick response to reports, and training of healthcare workers in reporting are essential to the maintenance of active surveillance [7].

Smartphones are products of technological innovations that offer features essential to keeping pace with the propagation of disease. They are a) connected to the Internet, b) portable, and c) able to function in remote locations [8]. For the public, this means that smartphones can be convenient sources of important health information and a point of contact to professional aid when necessary. For public health officers, the smartphone can enable near-instantaneous consolidation of reports made by themselves or by the public. Using the smartphone based surveillance applications, the accelerated transmission of information can shorten response times and provide authorities with a more accurate picture of disease situations for intervention planning and execution [9].

In Sri Lanka, mobile penetration rates have risen from 96% in 2012 to 126% in 2017 [10]. To capitalise on this, an integrated digital surveillance tool – Mo-Buzz – was developed and launched in 2013 to help mitigate the spread of dengue, details of which can be found in Lwin et al. [11] and below in Operating Environment of the Pre-Epihack existing Mo-Buzz System and Response and Challenges when Implementing Mo-Buzz PHIs and Mo-Buzz Public Section. Briefly, the application was first designed for public health inspectors (PHIs) who work on the frontline to assist them in educating the public, mapping potential dengue hotspots through the Global Positioning System (GPS), and reporting potential dengue fever cases. A second version was later piloted with the public to tap on crowd-sourced surveillance, and enabled PHIs to report potential breeding sites, contact health authorities directly, access educational material on dengue prevention, and get notified when outbreaks occur [11]. Table 1 illustrates in brief the workings of the original paper-based system in Sri Lanka and the direct improvements targeted with the creation of Mo-Buzz PHI and Mo-Buzz Public.

**Table 1:** Direct improvements on dengue surveillance targeted to be achieved by Mo-Buzz [9]

Function	Challenges	Intervention (Mo-Buzz)
Surveillance	- Paper reporting forms posted to Colombo Municipal Council (CMC) for consolidation and re-posted to Ministry of Health (MOH) by patient region - Dengue cases marked on physical map in MOH office-colour coding map with stickers is cluttered and time consuming	- Digital forms automatically sent to relevant - GPS geotagging of dengue sites accessible by all PHIs using Mo-Buzz, digitally colour coordinated by action.
Health Communication	- PHIs use paper leaflets/materials to inform public. Costly and time consuming to produce and outdated - Public have to seek out dengue information through traditional methods	- Educational materials assist PHIs to educate public - Public can access updated information on outbreaks - PHIs can respond to public queries more quickly and efficiently
Civic Engagement	- PHIs manually discover potential dengue cases through hospitals and visit public. Public are not actively seeking to notify of dengue cases	- Public can report location of breeding sites and submit potential dengue cases to instantly notify PHIs to investigate

These improvements streamlined the system by accelerating information flow to relevant persons from taking 7–10 days using traditional paper based methods, to 2–3 days. The shorter response times mean that it is more likely that health authorities would be able to discover and deal with the dengue sites faster [12].

### Operating environment of the pre-Epihack existing Mo-buzz system

The current system for Mo-Buzz PHIs is an Android application (for tablets) that uses Eclipse with Android Development Tools (ADT) plugin as an integrated development environment (IDE) and Google Maps application programming interface (API). This version also includes a management portal web site that uses Eclipse as an IDE and the programming language Java. The web portal consolidates the information reported via the application from various inspectors and assists health officials in analyzing them.

The Mo-Buzz public system consists of three main components, namely: “Mo-Buzz Dengue” (Android application), “Mo-Buzz Dengue Management Console” (Web application) and “Colombo Municipal Council (CMC) Public Health” (Android application). These three applications synchronize with Mo-Buzz Public backend to complete the workflow of the system. Mo-Buzz Dengue Management Console is the main administration-panel for the whole system. Through this application, personnel at CMC can manage all the data, public users and PHIs. CMC public health is the android app used by PHIs to access public data and check any public claims of potential dengue infected areas by visiting, monitoring and confirming the reported sites. PHIs are expected to follow up on valid reports by the public and update its status.

Mo-Buzz mobile users are registered on the android platform that was developed using Java. The backend and the web-client run on a scripting language programmed for web development called PHP (Hypertext Preprocessor) and are hosted within Apache (an open-source cross-platform web server). JavaScript Object Notation (Json) (an application used when exchanging data between a browser and a server) is used for communication whenever necessary within the system. Most communication with mobile users is done through Representational State Transfer (RESTful), a software that provides interoperability between web systems, however in instances where backward compatibility with older mobile apps (versions which are on the android market) is needed, the broader architectural style that encompasses RESTful called “REST communication” is used. The system is compatible with Android version 4.0 and above, Apache 2.4.10, PHP 5.3, and MySQL 5.6.

### Response and challenges when implementing Mo-buzz PHIs and Mo-buzz public

Despite low initial uptake due to the application being a novel and daunting method for dengue surveillance among experienced PHIs, Mo-Buzz PHIs was eventually adopted by the majority of the PHIs once people got used to and word of mouth spread to others in the team. Eventually, the PHIs were receptive and gave positive feedback about the application, with 76% of PHIs within the CMC using the application [9]. However, some challenges like a slow response or lag in the application and inaccuracies in GPS coordinates persisted.

PHIs had continued to use their version of Mo-Buzz since 2013, which was last upgraded in 2014. However due to improvements in technology over time and an inability to provide both hardware and software updates, the PHI version of Mo-Buzz had become obsolete. To achieve a functional product that made sense within the ecology of Sri Lanka’s health structures, private corporations, educational systems and sociocultural context, the next Mo-Buzz version would require input, resources, and expertise from all these stakeholders in addition to the technical skillsets of IT developers. Nevertheless, it was valuable as one of the first evidence based participating surveillance tools, which had been shown to be effective in battling vector disease at a major city level [9].

With regards to the public version of the application, due to political turmoil during the launch and physical distance of the main project team from the local collaborators, Mo-Buzz Public’s development was stymied and had remained a pilot project. In a pilot assessment, it was found that some members of the public who used the application also sent ‘junk reports’ unrelated to dengue breeding sites (e.g. complaints about rubbish, roadkill, etc.) which hampered efforts to deal with authentic reports. These issues need to be highlighted and rectified in the newly developed Epihack version.

Therefore, the aim of this paper is to discuss the adaptation and further development of Mo-Buzz tool. The paper takes into account the challenges faced within the original application and incorporates current technological solutions to combat dengue via a health hackathon in Sri Lanka.

### The interdisciplinary intervention

**Fig. 1:**
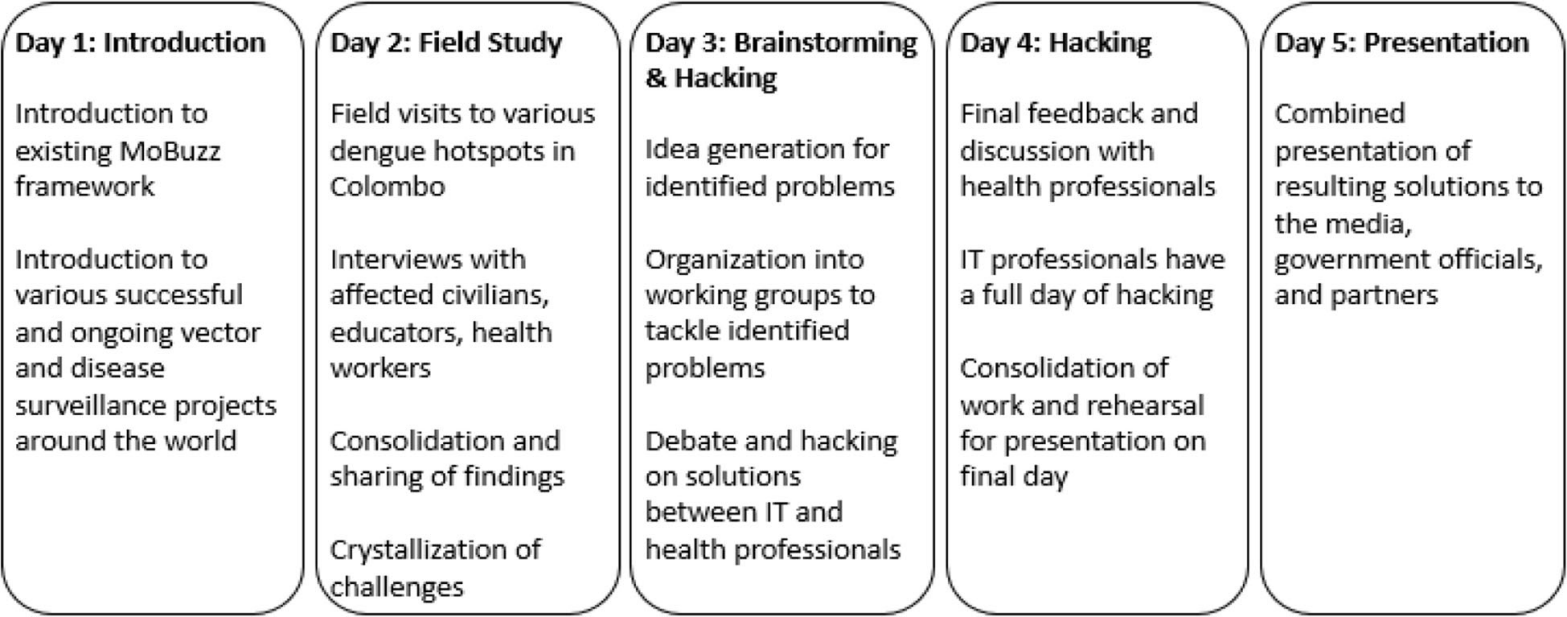
Outline of EpiHack™ Sri Lanka working model

With the learning experiences of the existing Mo-Buzz as a foundation, a five-day workshop called ‘EpiHack Sri Lanka’ was held in November 2017 (Fig. 1), supported by the Ending Pandemics program of the Skoll Global Threats Fund (a non-profit foundation based in San Francisco, CA) and the Colombo Municipal Council. It brought together IT and health experts from around the world to propose improvements on Mo-Buzz and create an integrated digital surveillance system to curtail the spread of dengue in Colombo.

The EpiHack model was developed by the Ending Pandemics program of the Skoll Global Threats Fund along with NGO and technology partners. EpiHacks – epidemiology hack-a-thons - are based on the hack-a-thon model popular in the field of computer science, where participants gather for an intensive workshop aimed at developing new software or technology tools. EpiHacks are collaborative events that convene participants from the technology, animal health, human health, design, and other sectors to develop new tools and solutions for combating infectious disease threats. All products and outcomes of EpiHack events are made free and open source for further adaptation and replication. In addition to Sri Lanka, EpiHacks have been held in Albania, Brazil, Cambodia, Laos, Myanmar, Tanzania, Thailand, Uganda, United States, and Vietnam.

EpiHack Sri Lanka was welcomed by various stakeholders (including the current Mayor of Colombo, advisor to the Sri Lankan Prime Minister and the National Dengue Unit) who saw the clear value of such efforts for the health of the citizens. Medical doctors and officials who will be the end users of the tool, worked together with IT professionals to provide valuable expertise and a thorough understanding of challenges, mitigating systems, and resources that were already in place.

Sixty health and IT experts from 11 countries attended the event, which involved information sharing and field visits to stimulate vibrant discussions to formulate creative ideas. Through various discussions, five groups were formed to each develop a different technical component of the surveillance system. The first day of the EpiHack was devoted to describing the challenges for dengue surveillance in Sri Lanka and ensuring that the technology partners understood the context of the challenge. As the week progressed participants engaged in site visits to areas prone to dengue and formed working groups around specific challenges that would be addressed in the next version of Mo-Buzz. Each group contained experts from the health and technology fields, and also consisted of the end users of the final application. Each group would regularly present their ideas and developments to the other groups to critically evaluate and ensure that each component was developed to the highest standard. At the end of the week each group presented their component to the stakeholders and discussed how to integrate these solutions. The ideas were evaluated by the stakeholders and end users who asked informed questions and highlighted any issues that could potentially arise from the fully developed tool, which is currently being developed.

## Implementation & Result: application development

### Prototype creation

**Fig. 2:**
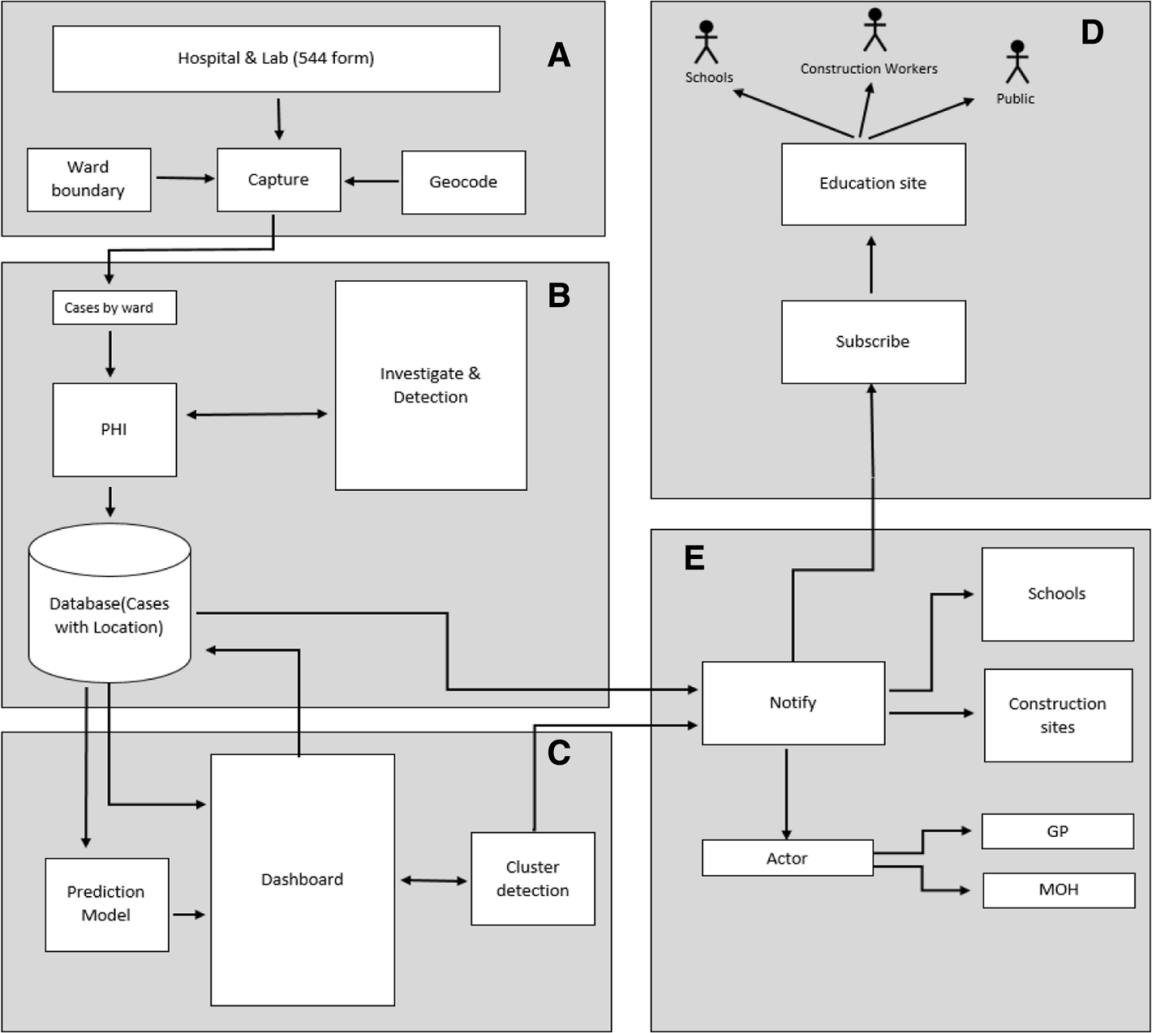
Flow chart illustrating synergies between proposed solutions for each collaborative component of the integrated system. **a** Hospitals and Labs; **b** PHI Consolidation and Action; **c** Analyses and Planning; **d** and **e** Targeted Dissemination and Education

We defined five major technical components of an overarching integrated surveillance system to address current gaps in dengue surveillance and meet stakeholder needs. Component A involved digitizing forms used by primary and secondary health care workers, component B consisted of creating a user-friendly system for the PHIs to gather dengue information, component C involved consolidating information from stakeholders and providing analytics, components D and E tailored educational materials and reporting systems for vulnerable groups and the general public. Details are provided in Fig. 2.

The technologies used in the system implementation can be categorized into two sections: the backend system and the frontend system. *Backend system:* The database system utilized MySQL, a database management system based on Structured Query Language, for both component A and B to remove the processing time needed to translate commands and retrieve data from multiple systems. MySQL is free to use and has been widely adopted among the developers, which made it easier and quicker for the team to obtain, set up and configure for the database system. This allowed more time for the development and refining of the frontend system. *Frontend system:* Component A used Hypertext Markup Language (HTML), a language for creating web applications, to develop web pages. Interfacing the web pages to the backend system utilizes a server scripting language and interpreter called PHP. By combining both HTML and PHP technologies together, the hospital forms were digitised effectively.

#### Component a (digitisation of hospital forms)

The first point of contact for potential dengue cases is usually the patient’s General Practitioner (GP) or local hospital. Patients provide blood samples for lab analysis to test for dengue. When a suspected dengue patient is reported to the hospital, GP, or lab, their information is collected via a paper form and sent to the CMC Epidemiological unit. The cases are categorized according to locality and sent to the relevant CMC office, where old forms are stored, taking up unnecessary room. Due to heavy workload and time-consuming paper processes of healthcare workers, reports are delayed and the CMC is sometimes not informed. Component A streamlined this process by digitizing the paper form and obtaining location details through geocodes that automatically categorize each locality. This would enable to forms to be received immediately by relevant bodies. A digitized system for hospitals and GPs is novel as it was not addressed with Mo-Buzz, which only focused on digitizing the PHI forms. Further advantages of digital forms include: 1) immediate automated error checking; 2) secure in a central database storage; and 3) minimal time between data entry and data analysis – which would reduce decision-to-intervention delay. Patient details from component A would be stored with PHI mosquito breeding site data from component B (below) to identify hotspots.

#### Component B (digital aid to public health inspectors (PHIs)

**Fig. 3:**
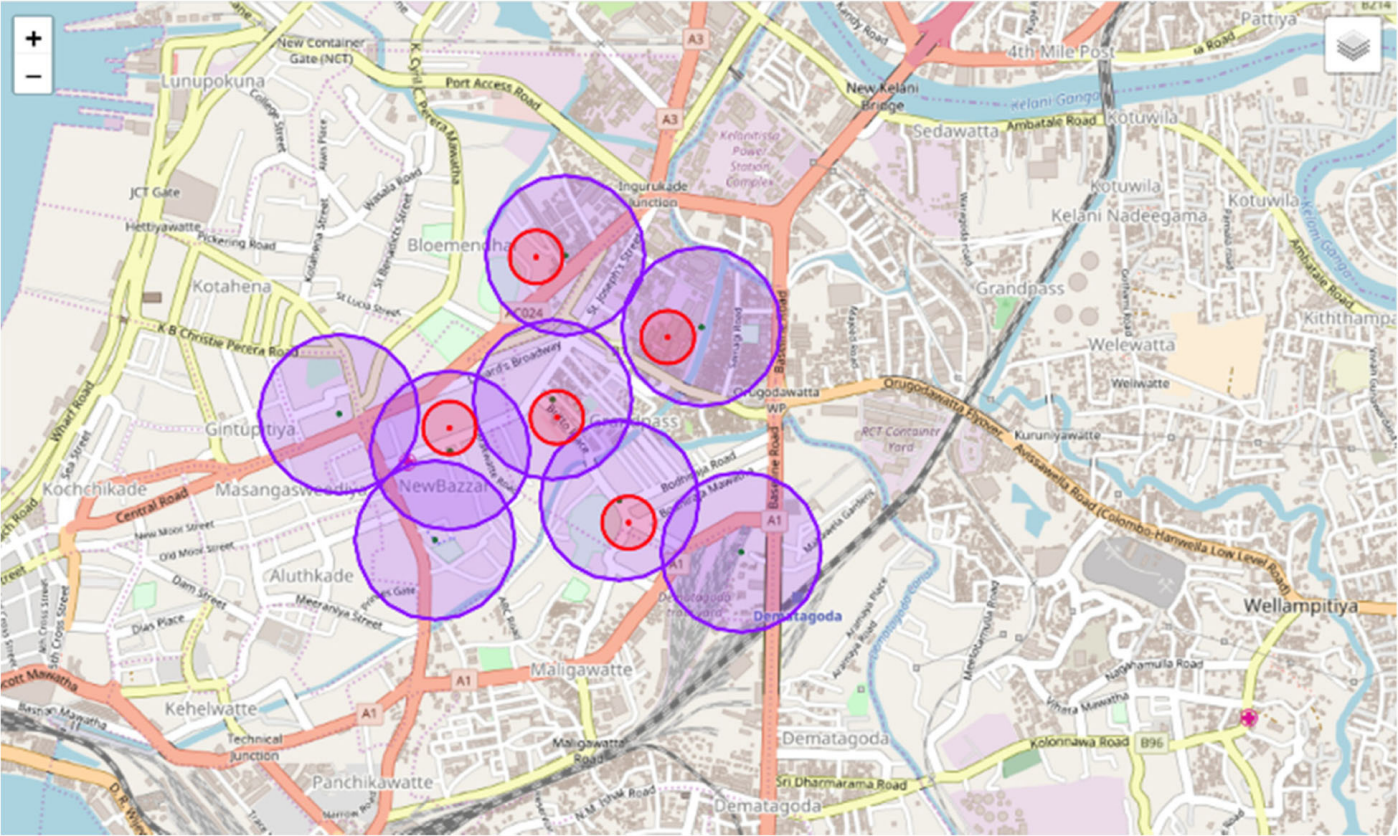
Red circles represent cases, purple circles represent mosquito breeding sites. The intersection between red and purple indicates elevated risk of dengue fever outbreaks. Maps used in the application are taken from OpenStreetMap®

In the current system, PHIs use paper-based methods of data collection and the outdated Mo-Buzz application, which are no longer time-efficient and adequate. Component B converted the paper methods documenting breeding sites into digital versions that automatically check for geo-location and data errors and collated all the information collected by PHIs into one database (via the updated mobile app). This would reduce total data collection time. This database acquires data collected from patients presenting with dengue from component A’s digital hospital forms and is supported by analytics from component C as shown in Fig. 3. The data is then presented in an interactive graphic which allows users to filter the information provided and collated in the system and facilitate meaningful analyses. Unlike the original Mo-Buzz application, which was only available via tablet, the new system has been designed for use on mobile phones, which all PHIs have access to and does not require the purchase of updated tablets. To capture and visualize data for the web-portal, Bootstrap, HTML, PHP, JavaScript and jQuery were used. For the mobile application, a cross-platform development tool called Iconic was used to ensure that the code could be used smoothly and efficiently to create both Android and iOS applications. Both the web-portal and the mobile application frontend connect to the backend where all of the logics and data are processed. To manage the logics, PHP was used, and to manage the data, MySQL was used to store all the data from the digitized documents. MySql and Iconic (prototype only) were used to develop the backend system. All the software was used in line with the other components to ensure the seamless integration of the final product with other developments to create a multifaceted system that built on the original Mo-Buzz by digitizing and networking a wider range of processes involved in dengue prevention.

#### Component C (data consolidation and analytics)

Currently, data obtained from local stakeholders are recorded on paper, filed together, and stored in physical filing units. The dengue hotspots are mapped manually by wards on large paper maps in each of the CMC ward offices. The component C prototype consolidated all the information from PHIs and relevant stakeholders into a dashboard using location-based clustering. This function would analyze the connection between breeding sites and cases and assist the CMC in prioritizing their responses based on risk modelling. This could help health inspectors prioritize the areas with the highest risk for their most immediate action. This could also increase the accuracy and efficiency of information sharing between wards by maintaining a centralized record of cases that is accessible to all relevant health officers.

Component C focused on the frontend system and drew on data from other components. The technologies used to create this utilized OpenStreetMap® and Leaflet.js. Leaflet is a JavaScript library for creating mobile-friendly interactive maps and was the core technology. OpenStreetMap® is an open API service that can create editable maps licensed under the Open Data Commons Open Database License (ODbL). Six layers of the application were developed shown in Fig. 3. The first layer of the application used OpenStreetMap® to visualize a map of Colombo. The second layer focused on highlighting areas at the most risk if an outbreak occurred by using models to calculate risk values using population density, building frequency, areas of water and other factors associated with the presence of mosquito breeding grounds. This layer’s boundaries were based on Colombo’s sub-district boundaries. The third layer focused on mapping specific high-risk areas such as the location of schools and construction sites. The fourth layer of the application mapped reported dengue cases taken from groups A and B. The fifth layer of the application mapped the recent history of mosquito breeding sites, so the health inspectors can keep track of the areas that need to be checked more regularly. The last layer of the application mapped the boundary of the outbreak and was detected by SatScan- a software program that can perform geographical surveillance of dengue to detect how dengue clusters are distributed over space and time to see if they should be identified as outbreak areas for intervention.

#### Component D (education, construction workers, GPs) and component E (education, school, public)

Both component D and E are mobile application user interfaces tailored to more adequately service to specific high-risk groups; namely schools, GPs, tourists, and the public. All versions included a map of nearby high-risk areas so that the user can be made aware of their immediate level of potential risk, reporting forms, reporting history frequencies by the user, their organization and their area, tailored educational resources, and a calendar for inspections, training and reporting.

Component D is an application with an intuitive interface and readily available educational materials to assist PHIs in engaging construction workers and managers at worksites, which commonly become dengue hotspots. Currently, construction sites appoint an officer to ensure workers check and keep areas clean and dengue free. If a worker is suspected of having the virus, a paper record is filled in and the CMC should be notified. Leaflets on how to prevent dengue are also handed out to workers. PHIs make regular inspection of the construction sites to check for mosquito breeding areas and proper implementation of prevention measures. If the site fails to apply adequate dengue prevention measures, they could be fined and taken to court. Despite this, many construction sites still fail to ensure that areas are kept clean and therefore remain hotspots for mosquito breeding grounds.

**Fig. 4:**
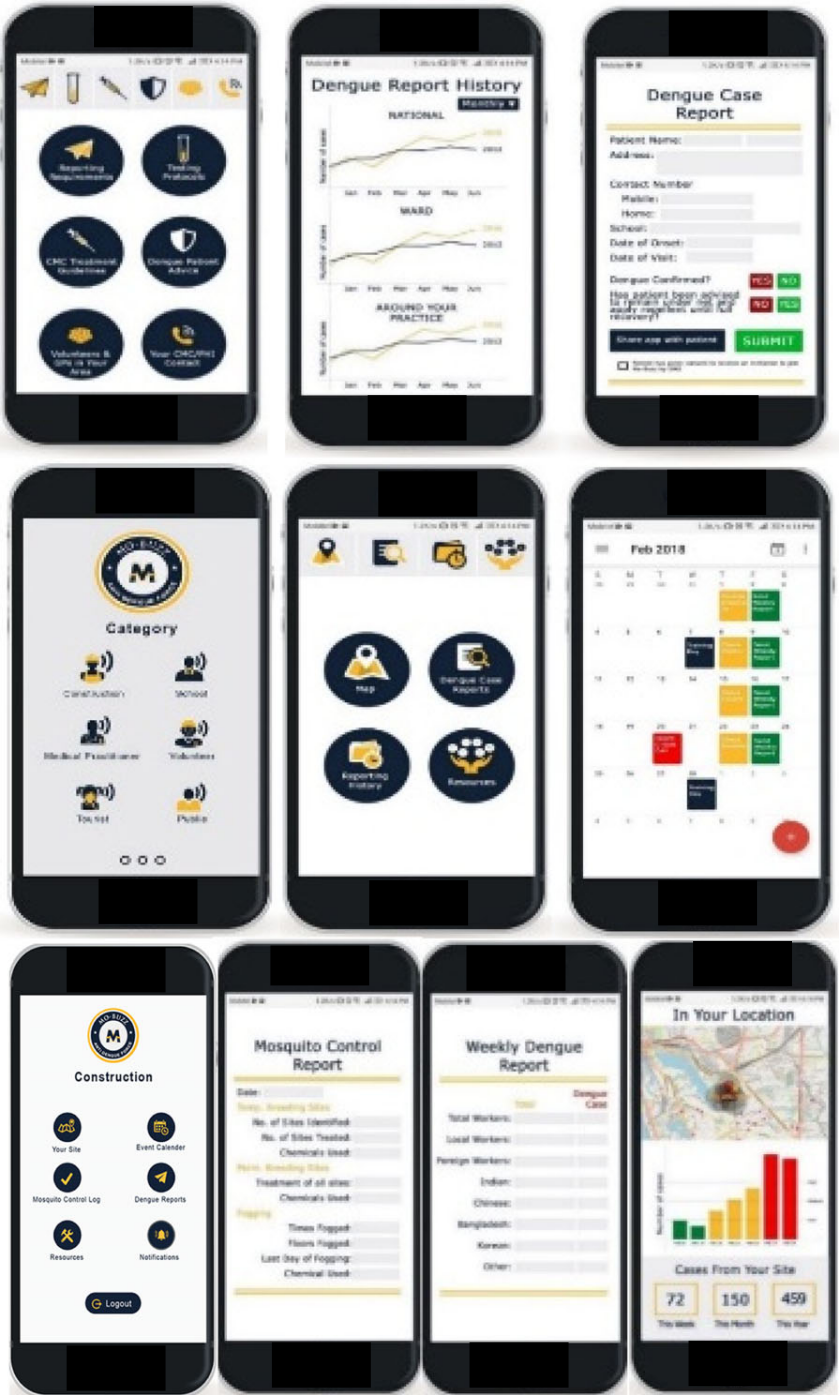
Illustration of simple user interface design for easy navigation and engagement for construction workers, GPs and the general population

The new application included ideas to create roles amongst construction workers for checking breeding sites, to roster these duties, and set calendar reminders for inspections, training ideas, case logging, and a weekly digitized report form to make reporting to the CMC quicker and easier (Fig. 4). The application also included educational materials and a hotspot map to warn of potential dengue cases in the surrounding area. As construction workers are predominantly from China and Sri Lanka, the option of having language support for the app in Mandarin and Sinhalese was proposed. Setting up a permanent banner at a prominent location on-site was also suggested to complement use of the application.

An interface was also developed and tailored towards GPs, with a digitized dengue reporting form, CMC guidelines for treatments, how to advise patients on protecting themselves from dengue, and direct contact to CMC (Fig. 4). GPs may also examine case histories by practice, ward and nation-wide to see how their area and practice fares compared to others. The development of an application greatly reduces the hassle associated with the current paper-based system. It is hoped that this digital system would increase GPs motivation for diligent reporting of dengue cases to CMC.

Component E is tailored towards schools and the public. Currently, schools also utilize paper-based educational material. Checklists are distributed to students to encourage proper procedures for reducing mosquito sites in schools and homes. Some schools have a dengue task force led by teachers and parents to reduce and prevent dengue breeding sites at school. Schools should report any potential dengue cases to the CMC and fill out a paper report, though underreporting also occurs. The CMC conducts monthly fogging and inspections of all schools’ premises and schools can opt to conduct more regular fogging or other preventative methods. Residential areas are also regularly fogged and CMC puts up educational posters in high-risk areas. However, residents are often resistant to their homes being labelled as a high-risk and do not want the fact advertised. This hints at some underreporting from residents as well because they want the ‘high-risk’ label to be removed quickly.

**Fig. 5:**
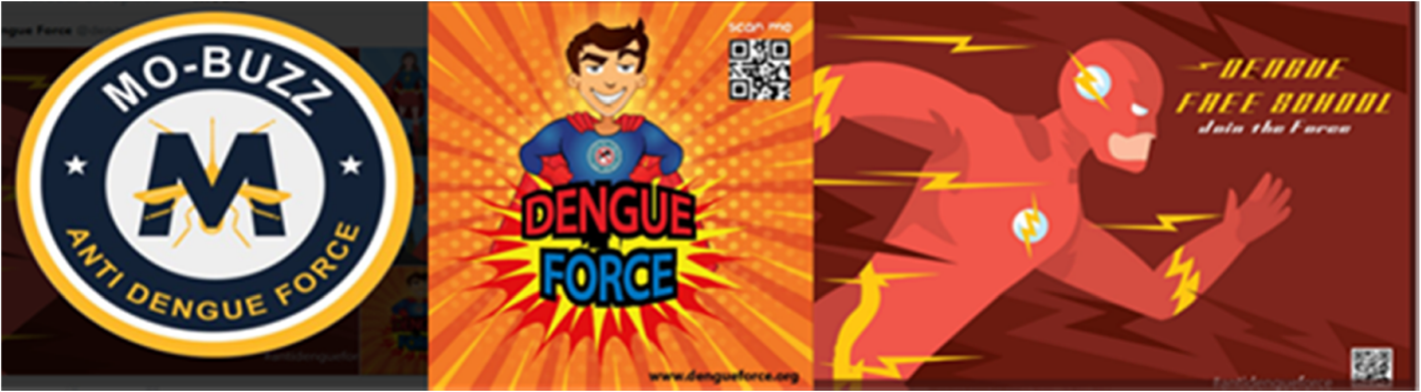
Eye-catching material to interest children and the general public to get involved were designed. Note: all figures included in this paper have been created by and belong to us

Schools have been identified as frequent hotspots for dengue fever transmission. The application was designed to assist PHIs in engaging children, teachers and the general public in their homes. Aside from the standard features of calendars, reporting, hotspot map, and push notifications, a social media presence was also created in the form of a Facebook page, YouTube videos and Twitter account to post educational materials on prevention methods. By using characters such as superheroes, the team attempted to make the material more appealing to children in order to increase the likelihood that they will engage with the application and become more motivated to assist in combating dengue (Fig. 5).

Each Component was routinely evaluated by the end users as the developments proceeded during the event. After the components were developed, the end users and stakeholders gave their inputs before the integrated prototype was developed. All the material created during the Epihack Sri Lanka are currently open source and had been uploaded into Github after the event. At the point of writing, the prototype surveillance system has been developed into a fully working system called ‘Mo-Buzz+’ for adoption by relevant users with only the final graphical user interface aspects needing to be completed. Meteorological and entomological data have also been incorporated into the predictive model to create a more precise estimate of dengue spread.

Currently, the prototype has gone through initial evaluation by 16 end users including doctors, public health inspectors and general users. The application has been received positively, with the users stating that it was easy to use, with less steps to complete each task and easier to search for patients. The users were pleased with the option of using the application on either tablet or mobile-unlike with the original Mo-Buzz which was only designed for tablet use. The analytics were well received, allowing users to compare various data. The users suggested updates in wording and some development of the user interface to make the appearance more appealing, which is now being worked on. Overall, they all liked the system and were confident that it can assist in cutting down time to help save lives and reduce the spread of the disease to others.

In recent years, with the advancement of wireless technology and mobile phones there have been numerous applications developed in healthcare, however Mo-Buzz+ is unique in its integrated approach. Many of the current infectious disease surveillance applications either solely look at educating and informing, such as the World Health Organisation’s Zika application (https://www.who.int/risk-communication/zika-virus/app/en/) or focus on one of the facets of the Mo-Buzz+ system such as mapping technology [13] or reporting survey [14, 15]. By integrating both the public, health authorities and other stakeholders into one application, the flow of communication, actions can be streamlined and the efficiency can be increased.

The hackathon event brought together experts in information technology to develop the system, as well as health experts and end users to provide expertise in the features requires to make the application effective. Initial evaluation of the final prototype suggests that by bringing together experts from different fields into one intense event can help in accelerating the development of a tool to combat dengue thereby addressing the challenges of the previous system. Creating a multifaceted, integrated system by improving the original Mo-Buzz application will hopefully streamline dengue surveillance to reduce the disease burden.

## Future development and conclusion

Ongoing discussions are being held with stakeholders to ensure better uptake of the application and motivate users to use the system. Once the prototype has been operationalized next year, a pilot test and a usability survey will be conducted to ensure the system meets the needs of the users to a high standard. The implementation of the system and a full release will then commence for Colombo and potentially the rest of Sri Lanka. If successful, the system can be adapted to other emerging infectious diseases to empower health authorities and the public to reduce disease burden globally.

The aim of this paper was to discuss the further development and adaptation of Mo-Buzz that integrated ideas from around the world using new technological solutions to create a novel, updated application during a hackathon event. By creating an integrated tool for the public and health authorities; the application could potentially accelerate the flow of information and enhance fast track surveillance of dengue with the ultimate goal of reducing the spread of the disease. This method of the development of health communication applications by bringing together experts from various fields could be increasingly utilised in the future to obtain a myriad of ideas to create technological solutions.

## Availability and requirements

The basis for development is available through github, but we are currently developing the application for a full release this year and the source code will be made available soon.

## Data Availability

The datasets used and/or analysed during the current study are open source and are available via Github. The github links for each groups source codes are: Group A Code https://github.com/Dinusha101/epihack2017-fighdengu-a Group B Code https://github.com/vajira-epihack2017/team-b Group C Code https://github.com/pphetra/dragon_ui/ Group D Code https://github.com/Sumudu-Sahan/dengueforce_epihack Group E Code https://github.com/dinukadesilva/epihack-2017

## Abbreviations

ADT: Android Development Tool
API: Application Programming Interface
CMC: Colombo Municipal Council
GP: General Practitioner
GPS: Global Positioning System
IDE: Integrated development Environment
MOH: Ministry of Health
ODbL: Open Database Library
PHI: Public Health Inspectors
